# Baseline Sensitivity of *Echinochloa crus-galli* (L.) P.Beauv. and *Leptochloa chinensis* (L.) Nees to Flusulfinam, a New 4-Hydroxyphenylpyruvate Dioxygenase (HPPD)-Inhibiting Herbicide in Rice, in China

**DOI:** 10.3390/plants14101425

**Published:** 2025-05-09

**Authors:** Zihao Li, Xinyu Sun, Shuo Yu, He Sun, Lei Lian, Xuegang Peng, Tao Jin, Weitang Liu, Hengzhi Wang

**Affiliations:** 1 College of Plant Protection, Shandong Agricultural University, Tai’an 271018, China; 15615486737@163.com (Z.L.); shuoyu_0323@163.com (S.Y.); 13210112668@163.com (H.S.); liuwt@sdau.edu.cn (W.L.); 2 Shandong Province Higher Education Provincial Key Pesticide Toxicology and Application Technology Laboratory, Tai’an 271018, China; 3 Qingdao KingAgroot Crop Science Groups Co., Ltd., Qingdao 266000, China; lianlei@kingagroot.com (L.L.); pengxuegang@kingagroot.com (X.P.); jintao@kingagroot.com (T.J.)

**Keywords:** barnyard grass, Chinese sprangletop, flusulfinam, dose–response assays

## Abstract

Flusulfinam is a 4-hydroxyphenylpyruvate dioxygenase (HPPD)-inhibiting herbicide applied post-emergence (POST) to control *Echinochloa crus-galli* (L.) P.Beauv., *Leptochloa chinensis* (L.) Nees, *Digitaria sanguinalis* (Linn.) Scop. and other annual weeds in directly seeded and transplanted paddy fields in China, registered in September 2024. Notably, compared with other HPPD inhibitors in rice, flusulfinam exhibits consistently high safety in both japonica and indica rice varieties. Meanwhile, flusulfinam has no target-site cross-resistance with traditional acetolactate synthase (ALS)-inhibiting, acetyl-CoA carboxylase (ACCase)-inhibiting, and auxin herbicides. Moreover, as the only heterocyclic-amide-structured herbicide in the HPPD inhibitors, it poses a low risk of metabolic cross-resistance with the other HPPD inhibitors, making it a promising candidate for managing herbicide-resistant weeds in rice fields. In this study, the baseline sensitivity to flusulfinam of *E. crus-galli* and *L. chinensis* in paddy fields in China was established using dose–response assays between June and October 2023. Thirty-nine populations of *E. crus-galli* and forty-three populations of *L. chinensis*, collected from rice fields across various major rice-producing regions in China, exhibited susceptibility to flusulfinam. The GR_50_ values ranged from 0.15 to 19.39 g active ingredient (a.i.) ha^−1^ for *E. crus-galli* and from 7.82 to 49.92 g a.i. ha^−1^ for *L. chinensis*, respectively, far below the field recommended rate of flusulfinam. Meanwhile, the GR_50_ values of *E. crus-galli* and *L. chinensis* to flusulfinam were both distributed as a unimodal curve, with baseline sensitivity (GR_50_b) of 6.48 g a.i. ha^−1^ and 22.38 g a.i. ha^−1^, respectively. The SI_50_ value showed 129.27-fold and 6.38-fold variability in flusulfinam sensitivity among the 39 *E. crus-galli* field populations and 43 *L. chinensis* filed populations, while the variability declined to 2.99-fold and 2.23-fold when the SI_50_b value was used. This study substantiated the efficacy of flusulfinam against *E. crus-galli* and *L. chinensis* in Chinese paddy fields and furnished a benchmark for monitoring temporal variations in the susceptibility of field populations of *E. crus-galli* and *L. chinensis* to flusulfinam.

## 1. Introduction

Rice (*Oryza sativa* L.) is a paramount food crop globally, especially in Asia, where it has been a dietary staple for centuries [1]. In China, rice serves as a staple food for more than 65% of the population, playing a crucial role in both food production and consumption among the Chinese populace [2]. Among the various modes of rice cultivation, rice direct seeding has a millennia-old history [3], offering benefits such as labor and cost savings, as well as increased yield [4,5]. In recent years, driven by a shortage of rural labor, rice cultivation methods have increasingly shifted towards lighter and simplified approaches, such as direct seeding rice. This trend has gained rapid momentum, particularly in the regions of Jiangsu and Zhejiang Provinces, China [6]. Currently, the proportion of direct-seeded rice cultivation areas in Jiangsu Province exceeds 20% of the total rice cultivation area. However, weeds persist as a challenging issue in the production and management of direct-seeded rice, posing a significant threat to achieving high-quality and high-yield harvests. Thus, effective weed control measures are paramount for optimizing the cultivation of direct-seeded rice fields [7]. However, the chemical control of weeds in rice fields, especially in direct seeding rice fields, is becoming increasingly difficult in China, mainly facing two major problems: firstly, there are many weed species with high occurrence density [8], among which the occurrence density of *Echinochloa crus-galli* (L.) P. Beauv. and *Leptochloa chinensis* (Linn.) Nees are absolutely dominant [9,10,11,12]. At the same time, the variety of weeds leads to community diversity [13,14], making it difficult for the current conventional herbicides to meet the needs of weed control. The second issue is the continuous spread of weed resistance, mainly manifested in the increasingly prominent resistance issues of widely used herbicides such as acetolactate synthase (ALS, EC 2.2.1.6) inhibitors, acetyl-CoA carboxylase (ACCase, EC 6.4.1.2) inhibitors, and auxin herbicides in rice fields, often leading to the failure of weed control [15,16,17,18,19,20,21,22]. With the emergence of the two major problems above, the phenomenon of overuse and increased rates of herbicides in rice fields will become more obvious, and the problem of herbicide damage will become more serious than before, which requires the use of safer and more efficient herbicides [23]. From the current development trend, the problem of resistant weeds will become a huge challenge for food safety production in rice fields, especially in direct seeding rice fields. Therefore, developing efficient and safe herbicides with new modes of action in rice fields is an effective means to solve the problem of resistant weeds.

Among the various known modes of action of herbicides, 4-hydroxyphenylpyruvate dioxygenase (HPPD, EC1.13.11.27) inhibitors stand out as a relatively recent addition to the commercial herbicide arsenal [24] and have become more popular and prevalent in recent years. By inhibiting the catalytic activity of HPPD, they block the conversion of 4-hydroxyphenylpyruvate (HPPA) to homogentisic acid (HGA), consequently disrupting the usual synthesis of plastoquinone (PQ) and α-tocopherol [25]. Plastoquinone (PQ) serves as a crucial cofactor for phytoene desaturase (PDS, EC1.3.5.6), a pivotal enzyme in the carotenoid biosynthesis pathway [26]. The absence of PQ hampers catalytic activity of PDS enzyme, thereby disrupting carotenoid biosynthesis [27]. Concurrently, α-tocopherol plays a role in neutralizing excessive peroxides and reactive oxygen species (ROS) triggered by intense light in plants [28,29]. The surplus ROS and carotenoid depletion result in chlorophyll and photosynthetic membrane degradation [30], as well as chlorosis (bleaching) in the meristematic and newly developed tissues of sensitive plant, ultimately culminating in plant death. Due to its complex plant toxicity mechanism, the resistance risk of HPPD-inhibiting herbicides is much lower than that of single-site inhibitors such as ALS- and ACCase-inhibiting herbicides [24].

There is no cross-resistance between HPPD inhibitor herbicides and the commonly used ALS-, ACCase-inhibiting, and auxin herbicides in rice fields, which can be used to solve the target resistance of weeds in rice fields. At the same time, the novel chemical structure also gives herbicide molecules the potential not to become substrates for enzymes in metabolically resistant weeds, thereby enabling herbicide compounds to have the potential to control metabolically resistant weeds. At present, the HPPD-inhibiting herbicides used in paddy fields in China mainly include mesotrione, benzobicyclon, tefuryltrione, and tripyrasulfone, but these four herbicides are difficult to promote due to their safety and herbicide spectrum limitations. For example, mesotrione has poor safety for rice and is only applied using the herbicidal soil method in rice transplanting fields [31,32]. Benzobicyclon has high efficacy against *Scirpus juncoides* Roxb., but poor efficacy against *E. crus-galli*, and is only used in rice transplanting fields [31,33,34]. The efficacy of tefuryltrione against *E. crus-galli* over the 2-leaf stage is poor, and it was limited to application with the herbicidal soil method in rice transplanting fields [31]. Although tripyrasulfone can be used to control *E. crus-galli* and *L. chinensis* in direct seeding rice fields, its safety varies among different rice varieties, with lower safety for indica rice and a risk of herbicide damage [35,36,37].

**Figure 1:**
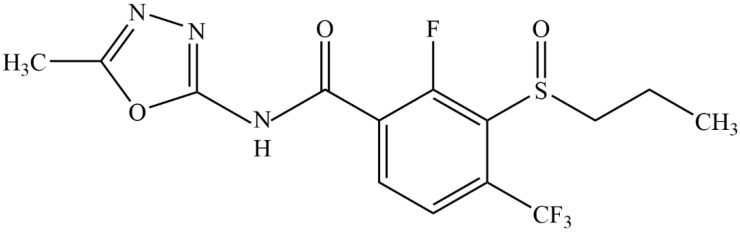
Chemical structure of flusulfinam.

Flusulfinam (Figure 1; Code Name: QYR601; CAS Registration Number: 2428458-82-4; Molecular formula: C_14_H_13_F_4_N_3_O_3_S; Benzamide, 2-fluoro-N-(5-methyl-1,3,4-oxadiazol-2-yl)-3-(propylsulfinyl)-4-(trifluoromethyl)-) is a new generation of HPPD-inhibiting herbicide in rice fields developed by Qingdao Kingagroot Crop Science Groups Co., Ltd. (Qingdao, China) and was granted initial registration in Cambodia (http://www.maff.gov.kh/ accessed on 21 March 2025). Preliminary studies have shown that flusulfinam possesses high post-emergence (POST) herbicidal activity against *E. crus-galli*, *L. chinensis*, *Digitaria sanguinalis*, as well as some broadleaf weeds and cyperaceae weeds. Different from other HPPD-inhibiting herbicides used rice fields, it is extremely safe for rice and meanwhile, there is no difference in safety between japonica and indica. Especially, flusulfinam is a chiral amide herbicide containing sulfoxide as the chiral center, with two enantiomers, R-(+)- and S-(−)-flusulfinam, which possess different bioactivity, toxicity, and degradation in paddy soils. The R-flusulfinam shows higher herbicidal activity against weeds like *E. crus-galli* and *D. sanguinalis* but is more toxic to algae (*Selenastrum capricornutum*), while the S-flusulfinam is more toxic to zebrafish larvae (*Danio rerio*). Degradation in paddy soils is enantioselective, with S-flusulfinam degrading faster in aerobic conditions and R-flusulfinam in anaerobic soils from certain regions. Degradation is influenced by soil organic matter and pH, with microbial communities playing a key role. Two hydrolysis metabolites, M299 and M100, were identified. Overall, R-flusulfinam is more effective for weed control with lower toxicity to fish, highlighting its potential for targeted use. However, S-flusulfinam exhibited preferential transport to the shoots and the whole plant and were preferentially degraded [38,39].

Although HPPD inhibitors have been deemed low-risk herbicides in terms of resistance evolution, their application has led to the gradual emergence of resistance issues. Certain weeds have developed resistance to HPPD-inhibiting herbicides and four dicot weed species are currently resistant to HPPD inhibitors, including three weed species in the Amaranthaceae family, including *Amaranthus palmeri*, *Amaranthus retroflexus*, and *Amaranthus tuberculatus* [40,41,42,43,44,45,46], which were found in the US states of Kansas, Nebraska, Wisconsin, North Carolina, Illinois, and Iowa, as well as in Ontario, Canada. Another case of resistance to HPPD inhibitors was the *Raphanus raphanistrum* L., found in Western Australia [47,48]. In particular, it is reported that *L. chinensis* in rice fields of Hunan Province, China, first evolved resistance to tripyrasulfone [49]. The cases above suggest that widespread and sustained use of flusulfinam must eventually lead to weed resistance to the herbicide.

Therefore, in order to quickly clarify the current resistance status of weeds to HPPD-inhibiting herbicides in different regions and predict the development trend of resistant weeds (especially some low-level resistance such as non-target resistance) [50], it is necessary to conduct real-time resistance monitoring of weeds in the rice fields, providing a theoretical basis for weed resistance management [51,52]. Establishing a comprehensive system for monitoring weed resistance and conducting early resistance surveillance is pivotal, with sensitivity baselines serving as the cornerstone for resistance monitoring and the benchmark for evaluating weed resistance [53]. When sensitivity data for a specific herbicide are derived from weed populations that have not been previously exposed to that herbicide or possess the same mode of action as previously applied, these data can be deemed as baseline sensitivities [54]. At present, no baseline data on the sensitivity of any weed species to flusulfinam has been reported.

*Echinochloa crus-galli* and *L. chinensis* are harmful weeds in rice fields, and the problem of multi-resistance of them is becoming more and more serious. Flusulfinam possesses enormous potential for application in paddy fields. In order to scientifically and rationally use flusulfinam, delay the development of resistance, and extend the service life of flusulfinam, sensitivity data are essential information. Therefore, the aim of this study was (1) to evaluate the efficacy of flusulfinam against *E. crus-galli* and *L. chinensis* by assessing the sensitivity of 39 *E. crus-galli* populations and 43 *L. chinensis* populations collected from rice fields in China, and (2) to establish baseline sensitivity to monitor the response of *E. crus-galli* and *L. chinensis* populations to flusulfinam in rice fields in the future.

## 2. Materials and Methods

### 2.1. Plant Materials

**Figure 2:**
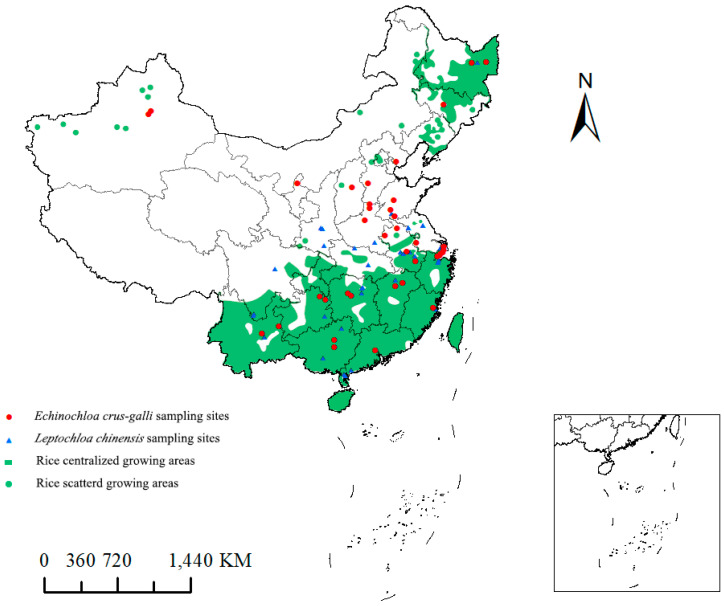
Distribution of 39 *E. crus-galli* populations and 43 *L. chinensis* populations collected from paddy fields in China.

From 2018 to 2022, seeds of 39 different *E. crus-galli* populations and 43 different *L. chinensis* populations were collected from the rice fields in regions of China for large-scale rice cultivation (Figure 2). For each population of *E. crus-galli* and *L. chinensis*, mature seeds were randomly gathered from more than 200 plants and then mixed evenly. After collection, the seeds were dried and stored in a kraft paper bag at room temperature (20–25 °C). At the same time, during seed collection, inquiries were made with farmers regarding the history of herbicide use in the collected fields to ensure that no HPPD-inhibiting herbicides had been used before in the field.

The *E. crus-galli* and *L. chinensis* seeds, exhibiting full and uniform grains, were placed in a culture dish covered with two layers of filter paper (Whatman No.1, Maidstone, UK). Subsequently, 8 mL of sterile water was added to maintain moisture levels. The seed-containing dishes were then transferred to an artificial climate chamber (Model RXZ-500, Ningbo Jiangnan Instrument Factory, Ningbo, China), set at a constant temperature of 25 °C, with photoperiod of 12/12 h, relative humidity 75% to accelerate seed germination. When the roots of germinated seeds became visible, twenty seeds were sown into a 12 cm-diameter plastic pot filled with loam soil (organic matter of 15.79 g kg^−1^, pH of 7.67, and 14.28/2.0/23.15 g kg^−1^ of N P K) passed through a 3 mm sieve before use. The pots were placed in an artificial climate chamber (500 μE m^−2^ s^−1^ photosynthetically active radiation, 32/24 °C, 12/12 day/night, and 60% relative humidity) for *E. crus-galli* and *L. chinensis* plant growth [55]. When the weed plants reached the 1–2 leaf stage, the seedlings were thinned, and 15 uniformly sized seedlings were carefully selected and retained for further experiments.

### 2.2. Flusulfinam Dose–Response Assays

When the weed plants reached the 4 leaf stage, a research track sprayer (Model ASS-4, National Agricultural Information Engineering and Technology Center of China, Beijing, China) equipped with a Teejet flat-fan nozzle (9503EVS, TeeJet Technologies (NINGBO) Co., Ltd., Ningbo, China) was used to conduct flusulfinam (6% oil dispersion, Qingdao Kingagroot Crop Science Groups Co., Ltd., Qingdao, China) POST application at 0, 1.88, 3.75, 7.5, 15, 30, and 60 g active ingredient (a.i.) ha^−1^, delivering a 450 L ha^−1^ spray of solution under pressure of 275 kPa. The untreated control was set for each weed population and treated with water. After application, the plants were transferred to the artificial climate chamber with the same conditions as described above. At 21 days after treatment (DAT), the weed seedling shoots from each pot were harvested and dried at 80 °C for 72 h in an air bellow (Model DHG9140A, Changzhou Noki Instrument Co., Ltd., Changzhou, China), and subsequently the dry weights were recorded. Each treatment was set up in four repetitions, and the entire experiment was conducted twice between June and September 2023.

### 2.3. Data Analysis

Analysis of variance was performed on data from the two repeated trials using SPSS software (version 22.0; International Business Machines Corporation, Armonk, NY, USA). The data were pooled together, as there was no significant difference between the same treatment (*p* > 0.05).

The GR_50_ values (the dose of flusulfinam that resulted in a 50% reduction in the growth) of each *E. crus-galli* and *L. chinensis* population were calculated with SigmaPlot software (version 13.0; Systat Software) according to the four-parameter loglogistic response equation [56]:

*y* = *c* + (*d* − *c*)/[1 + (*x*/*GR*_50_)*^b^*]

where *y* is the percentage of dry weight residue compared with untreated control, *x* is the flusulfinam dose, *c* is the lower limit, *d* is the upper limit, *b* is the slope at *GR*_50_. The normality of GR_50_ values was tested with Shapiro–Wilk test using OriginPro software (version 2017, OriginLab Corporation). The baseline sensitivity of *E. crus-galli* and *L. chinensis* to flusulfinam (GR_50_b) was the average of GR_50_ values of all populations; the SI_50_ values (sensitivity index at 50% growth reduction) of *E. crus-galli* and *L. chinensis* are shown as the ratio between the GR_50_ values of the most tolerant and the most susceptible populations; the SI_50_b values for *E. crus-galli* and *L. chinensis* were calculated as the ratio between the GR_50_ value of the least susceptible population and the baseline GR_50_ value [57].

## 3. Results and Discussion

**Figure 3:**
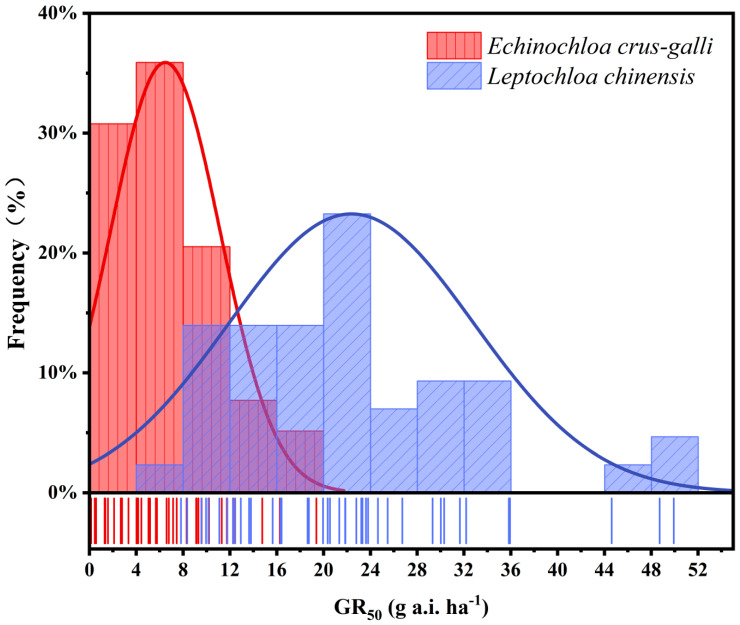
Frequency distribution of flusulfinam doses that caused a 50% growth reduction (GR_50_) for 39 *Echinochloa crus-galli* populations and 43 *Leptochloa chinensis* populations. Individual isolates are grouped in class intervals of 4 g a.i. ha^−1^.

*Echinochloa crus-galli* and *L. chinensis* pose significant weed control challenges in rice fields, with their distribution spanning across most regions worldwide [58]. Thirty-nine *E. crus-galli* populations and forty-three *L. chinensis* populations were collected in rice fields of different major rice-producing regions in China, and their GR_50_ values to flusulfinam are presented in Table 1 and Table 2, respectively, which can be used as an index to measure the sensitivity of weeds to herbicides [57]. The GR_50_ values of *E. crus-galli* to flusulfinam ranged from 0.15 to 19.39 g a.i. ha^−1^, and the GR_50_ values of *L. chinensis* ranged from 7.82 to 49.92 g a.i. ha^−1^, both of which were significantly lower than the flusulfinam field recommended rate of 90 to 120 g a.i. ha^−1^, proving that all tested weed populations of *E. crus-galli* and *L. chinensis* were sensitive to flusulfinam. The GR_50_ values of *E. crus-galli* and *L. chinensis* to flusulfinam were both distributed as a unimodal curve (Figure 3), and thus, the mean GR_50_ values of each population of *E. crus-galli* and *L. chinensis* were considered as their baseline sensitivity to flusulfinam. The GR_50_b values of *E. crus-galli* and *L. chinensis* were 6.48 g a.i. ha^−1^ and 22.38 g a.i. ha^−1^, respectively. Obviously, the baseline sensitivity of *L. chinensis* to flusulfinam was higher than that of *E. crus-galli*, indicating that *E. crus-galli* was more sensitive to flusulfinam than *L. chinensis*. As can be seen from Figure 3, the distribution of GR_50_ values of both *E. crus-galli* and *L. chinensis* skewed towards the more sensitive end, suggesting a low potential for resistance development in *E. crus-galli* and *L. chinensis* skewed to flusulfinam, although the frequency distribution of GR_50_ values does not necessarily indicate the absolute risk of weed resistance development [59].

The SI_50_ is a measure of response variability between weed populations [57]. Table 3 shows SI_50_ values of the present study, with a 129.27-fold difference in sensitivity for the *E. crus-galli* populations and a 6.38-fold difference in sensitivity for the *L. chinensis* populations, respectively. The much higher SI_50_ value for *E. crus-galli* than *L. chinensis* might indicate that the potential for *E. crus-galli* to develop resistance to flusulfinam is much higher than that for *L. chinensis* [55,60,61]. The differences in sensitivity to the same herbicide among different sensitive populations may be due to natural variation in herbicide sensitivity among populations [62,63,64] or may be due to differences resulting from repeated use of similar categories of herbicides (with similar chemical structures or similar modes of action) leading to selection pressure [65,66]. The populations of *E. crus-galli* and *L. chinensis* with the highest GR_50_ value may be in a higher selection stage for resistance to flusulfinam.

When using the GR_50_b value instead of the GR_50_ value of the most sensitive population to calculate the sensitivity difference index among populations, the sensitivity difference (SI_50_b) of different *E. crus-galli* and *L. chinensis* populations to flusulfinam decreased from 129.27-fold to 6.38-fold, and 2.99-fold to 2.23-fold, respectively. Therefore, establishing a sensitivity baseline should take into account the natural variability in sensitivity among different weed populations, and using the GR_50_b value may be a more practical parameter than using the GR_50_ value of the most sensitive population as the sensitivity baseline [57]. However, it is important to note that GR_50_ values are highly dependent on experimental conditions. Brent et al. [67] found that the GR_50_ value of *Abutilon theophrasti* Medicus to glyphosate ranged from 28 to 120 g a.i. ha^−1^ at different growth stages of the plant. Only under conditions where plant growth conditions (temperature, relative humidity, and light intensity), plant phenological stage, herbicide product used, and assessment time are consistent, do the obtained GR_50_ values have meaningful comparability.

As cases of cross-resistance and multiple resistances become increasingly prevalent, agricultural producers face limitations in their herbicide options. Currently, there are four documented instances of resistance to HPPD-inhibiting herbicide, which were all attributed to metabolic resistance [19]. Before the widespread development of herbicide resistance, proactive monitoring studies are crucial for detecting shifts in weed sensitivity to herbicides, which enables the timely implementation of strategies to mitigate or even prevent the development of resistance. In this study, sensitivity baselines of 6.48 g a.i. ha^−1^ 22.38 g a.i. ha^−1^ for *E. crus-galli* and *L. chinensis* in Chinese rice fields to flusulfinam that is vital for monitoring changes over time in the sensitivity of weed populations. Meanwhile, to delay the development of resistance and ensure the sustainable application of flusulfinam, it is crucial to reduce herbicide selection pressure. This can be achieved through the use of pre-emergence herbicides, rotation or mixing of herbicides with diverse modes of action, or the implementation of specific agronomic practice.

## 4. Conclusions

The baseline sensitivity to flusulfinam of *E. crus-galli* and *L. chinensis* populations collected from rice fields across China, expressed mean GR_50_ values, was 6.48 g a.i. ha^−1^ and 22.38 g a.i. ha^−1^, respectively. Meanwhile, there is a 129.27-fold difference in sensitivity for the *E. crus-galli* populations and a 6.38-fold difference in sensitivity for the *L. chinensis* populations, suggesting that naturally differences in sensitivity to flusulfinam do occur.

## Figures and Tables

**Table 1:** GR_50_ values of flusulfinam for each *Echinochloa crus-galli* population in rice fields in China.

Population	Sampling Sites	Latitude/Longitude	Flusulfinam (g a.i. ha^−1^)	
			^a^ GR50	^b^ SE
JX-1	Jiangxi Province	28.70° N/116.69° E	12.29	0.34
JX-2		28.54° N/115.94° E	10.21	0.58
JS-1	Jiangsu Province	34.72° N/116.93° E	2.79	0.25
JS-2		32.04° N/118.76° E	5.77	0.26
JS-3		30.47° N/118.36° E	6.59	1.21
JS-4		34.72° N/116.93° E	16.27	3.24
SH-1	Shanghai City	31.23° N/121.47° E	7.46	0.64
SH-2		30.74° N/121.34° E	19.39	1.88
SH-3		30.91° N/121.47° E	13.80	4.02
ZJ-1	Zhejiang Province	30.50° N/120.68° E	9.58	1.24
ZJ-2		30.67° N/121.01° E	5.04	1.33
ZJ-3		30.52° N/120.90° E	9.12	1.03
AH-1	Anhui Province	33.16° N/115.62° E	1.30	0.53
AH-2		33.63° N/116.98° E	4.02	0.67
AH-3		31.41° N/117.60° E	11.30	1.83
HLJ-1	Heilongjiang Province	46.80° N/130.37° E	4.17	0.24
HLJ-3		46.48° N/132.29° E	4.44	3.05
XJ-1	Xinjiang Uighur Autonomous Region	43.79° N/87.62° E	0.57	1.20
XJ-2		43.47° N/87.41° E	1.35	0.16
hn-1	Henan Province	36.10° N/114.35° E	1.58	0.45
hn-2		35.74° N/114.29° E	7.15	0.47
hn-3		34.74° N/113.61° E	2.72	0.35
NX-1	Ningxia Hui Autonomous Region	38.46° N/106.27° E	0.45	0.34
HB-1	Hebei Province	39.62° N/118.15° E	9.15	0.17
HB-2		38.04° N/114.50° E	4.07	0.39
JL-1	Jilin Province	43.90° N/125.32° E	6.78	0.27
YN-1	Yunnan Province	25.03° N/102.70° E	5.67	0.54
YN-2		25.68° N/104.26° E	14.76	2.49
FJ-1	Fujian Province	26.08° N/119.30° E	2.68	0.13
SD-1	Shandong Province	36.19° N/117.13° E	2.11	0.19
HLJ-2		35.40° N/116.59° E	11.73	0.95
SX-1	Shanxi Province	37.85° N/112.54° E	4.08	0.58
GD-1	Guangdong Province	23.15° N/113.27° E	6.59	0.35
HN-1	Hunan Province	28.34° N/111.20° E	1.58	0.45
HN-2		28.14° N/111.46° E	0.15	0.47
GX-1	Guangxi Zhuang Autonomous Region	24.39° N/109.53° E	9.31	1.11
GX-2		23.71° N/109.46° E	8.30	0.78
GZ-1	Guizhou Province	27.91° N/108.86° E	3.33	0.64
GZ-2		28.20° N/108.38° E	5.18	0.85

^a^ GR_50_ indicates the flusulfinam dose causing a 50% growth reduction in *E. crus-galli*, and was calculated according to a four-parameter log-logistic response equation: *y* = *c* + (*d* − *c*)/[1 + (*x*/*GR*_50_)*^b^*]. ^b^ SE indicates standard error of GR_50_ estimates.

**Table 2:** GR_50_ values of flusulfinam for each *Leptochloa chinensis* population in rice fields in China.

Population	Sampling Sites	Latitude/Longitude	Flusulfinam (g a.i. ha^−1^)	
			^a^ GR_50_	^b^ SE
AH-1	Anhui Province	30.92° N/118.34° E	48.72	4.30
AH-2		31.25° N/117.29° E	22.80	1.04
AH-3		31.22° N/117.23° E	10.17	0.65
AH-4		31.29° N/117.95° E	26.74	2.94
AH-5		31.47° N/116.94° E	21.84	3.14
FJ-1	Fujian Province	26.08° N/119.30° E	32.19	2.05
FJ-2		25.96° N/119.52° E	11.10	0.80
GD-1	Guangdong Province	21.64° N/110.92° E	30.03	0.92
GD-2		21.38° N/110.25° E	35.87	0.97
GD-3		21.19° N/110.39° E	13.79	0.97
GX-1	Guangxi Zhuang Autonomous Region	22.85° N/108.37° E	23.80	1.17
GX-2		25.27° N/110.30° E	18.75	5.97
HB-1	Hubei Province	30.68° N/113.50° E	12.26	2.12
HB-2		32.29° N/112.21° E	9.56	1.34
hn-1	Henan Province	32.60° N/114.39° E	31.65	5.32
HN-1	Hunan Province	28.64° N/112.61° E	12.45	1.35
HN-2		28.72° N/112.68° E	25.47	0.73
HN-3		28.25° N/112.56° E	18.63	0.85
JS-1	Jiangsu Province	33.47° N/119.80° E	44.62	4.67
JS-2		33.46° N/118.22° E	35.83	2.90
JS-3		33.46° N/118.22° E	12.93	0.97
JS-4		33.47° N/119.80° E	8.36	0.68
JX-1	Jiangxi Province	29.00° N/116.05° E	13.64	1.47
JX-2		29.04° N/116.06° E	20.35	0.68
SC-1	Sichuan Province	30.68° N/103.85° E	21.86	1.13
SC-2		26.69° N/101.85° E	49.92	4.89
SC-3		26.50° N/101.74° E	23.22	1.63
SD-1	Shandong Province	35.00° N/116.65° E	7.82	0.98
SH-1	Shanghai City	31.15° N/121.12° E	19.95	0.99
SH-2		31.22° N/121.54° E	30.31	0.92
SH-3		31.12° N/121.15° E	16.41	0.97
SH-4		31.21° N/121.54° E	11.78	0.93
SX-1	Shaanxi Province	32.70° N/109.03° E	15.65	0.55
SX-2		34.33° N/108.71° E	21.34	1.55
SX-3		34.27° N/108.96° E	20.55	1.45
SX-4		34.27° N/108.94° E	9.96	0.94
YN-1	Yunnan Province	24.67° N/102.91° E	24.64	2.89
ZJ-1	Zhejiang Province	29.99° N/120.58° E	18.74	6.46
ZJ-2		30.13° N/120.76° E	35.93	0.96
ZJ-3		29.97° N/120.60° E	23.62	0.75
ZJ-4		29.95° N/120.64° E	16.32	0.98
HLJ-1	Heilongjiang Province	46.65° N/131.17° E	23.32	1.35
GZ-1	Guizhou Province	26.44° N/108.71° E	29.32	2.34

^a^ GR_50_ indicates the flusulfinam dose causing a 50% growth reduction in *L. chinensis*, and was calculated according to a four-parameter log-logistic response equation: *y* = *c* + (*d* − *c*)/[1 + (*x*/*GR*_50_)*^b^*]. ^b^ SE indicates standard error of GR_50_ estimates.

**Table 3:** Indices of flusulfinam sensitivity of *Echinochloa crus-galli* and *Leptochloa chinensis*.

Weed Species	SI_50_ (g a.i. ha^−1^) ^a^	SI_50_b (g a.i. ha^−1^) ^b^
*E. crus-galli*	129.27	2.99
*L. chinensis*	6.38	2.23

^a^ the SI_50_ values (sensitivity index at 50% growth reduction) are shown as the ratio between the GR_50_ values of the most tolerant and the most susceptible populations; ^b^ the SI_50_b values were calculated as the ratio between the GR_50_ value of the least susceptible population and the baseline GR_50_ value.

## Data Availability

Data are available from the author.
